# Dynamic duos: learning to care as a pair in the biparental prairie vole (*Microtus ochrogaster*)

**DOI:** 10.3389/fnbeh.2025.1698616

**Published:** 2025-11-26

**Authors:** Taylor D. Hinton, Rebecca E. Waugh, Per B. Sederberg, Jessica J. Connelly, Allison M. Perkeybile

**Affiliations:** 1 Department of Psychology, University of Virginia, Charlottesville, VA, United States; 2 Program in Fundamental Neuroscience, University of Virginia, Charlottesville, VA, United States

**Keywords:** *Microtus*, maternal care, paternal care, prairie vole, biparental care, behavioral flexibility

## Abstract

**Introduction:**

A growing body of evidence shows that paternal care has long-lasting impacts on the social behavior of offspring, both in humans and other mammalian biparental species. However, fatherhood has historically been understudied and the dynamics of parental care adjustments based on their partner’s behavior remain unclear. This study investigates how individuals adjust parenting behavior based on their experience as part of a parenting dyad in the biparental prairie vole (*Microtus ochrogaster*).

**Methods:**

We investigated how prairie voles learn to be parents by observing how their parental care effort changes over two consecutive litters. The first litter represents a naive context while the second litter represents an experienced context.

**Results:**

On average, dyads provided 9% more care in the naive context than in the experienced context. Experienced mothers, as a group, tended to reduce care significantly, while experienced fathers did not. By comparing the correlation between mother and father care in the naive versus experienced contexts, we found that parental care became more negatively correlated following experience. Finally, we investigated whether the difference in the amount of care provided by each parent in the dyad in the naive context drives the observed changes in experienced parental behavior, and found that these differences significantly predict the likelihood of reducing or increasing parental care effort in the experienced context for both the male and female partner.

**Conclusion:**

Our results indicate that individual care behavior is adjusted based on the parenting effort of the dyadic partner. When only group-wise analyses are conducted, it appears that only mothers reduce care based on experience. However, through a dyadic-based analysis, we find that a larger difference in care between the two parents in the naive context corresponds to greater shifts in care by both parents in the experienced context. In sum, two patterns emerge in experienced parents that appear to improve parental care efficiency: (1) parents take on a more compensatory pattern of caregiving over time and (2) are able to adapt to initial differences in care such that investments in care become more balanced between mothers and fathers over time.

## 1 Introduction

Human parenting practices are highly unusual: unlike the majority of other mammalian species, both mothers and fathers, along with other adults, commonly serve in caregiving roles. Approximately 3%–9% of all mammalian species show evidence of social monogamy and father care toward offspring (Kleiman, 1977; Lukas and Clutton-Brock, 2013). It has been theorized that, while the lengthy nature of child development conveys a range of adaptive benefits that have allowed humans to flourish, the imposed costs to mothers are very high (Kleiman, 1977). These conditions produce adaptive pressure; to maintain higher overall fertility and enhance the likelihood of child survival, shared parental care by both mothers and fathers became a common human parenting scenario (Fletcher et al., 2015; Abed, 2025). Although cultural and environmental conditions have produced highly varied modes of living amongst different people, monogamous pair-bonding and father-care occur so commonly as to represent a human characteristic (Fletcher et al., 2015). However, the extent to which interacting environmental and genetic factors across the parent dyad contribute to parenting variability has been challenging to fully study.

With limited exceptions, given a safe and stable environment, parents generally invest sufficiently to ensure their children’s survival (Lu et al., 2023). Yet, within the constraint of a certain minimum investment, parenting behaviors and level of care can take many forms, with potential short and long term beneficial or deleterious impacts on the child. Unlike many other species, it has been argued that humans show a wide variety of specific parenting behaviors and styles, with relatively few predetermined “fixed action patterns,” rather behaviors are acquired through a combination of the experience of being cared for and the experience of caring (Hrdy, 2009). The tenor of this parental care can have many impacts. For example, parenting style has been linked to mental health outcomes in children and adolescents (Makwana et al., 2023) and even decades later, middle-aged and older adults may show improved health outcomes based on the parenting style they experienced when young (Rothrauff et al., 2009). The potential impact of these experiences reflects a combination of genetic and environmental factors, alongside a complex set of dyadic and multi-person relationships, including the parent-child dyad, the parent-parent dyad, and the interactions of these relationships with other family members, caregivers and the wider community (Branje et al., 2020).

The parental characteristics of the father-mother dyad emerge from the independent behaviors of each parent and how these traits interact with one another (Bodenmann, 1997; Feinberg, 2002; Falconier et al., 2015). The development of these behaviors is likely a dynamic process, reflecting each parent’s experiences with their own parents, with the other parent, and with their children. Relationships with contemporaries mediate individual parenting behavior (Belsky et al., 2005). Monogamous male-female partnerships, in which a pair-bonded couple produce and collectively provide care for genetically-related offspring, are common parenting structures (Fletcher et al., 2015; Abed, 2025). However, the role of the father in child rearing has only relatively recently become a focus of research interest (Taraban and Shaw, 2018). Paternal care can impact infant outcomes directly and indirectly. For example, a father may indirectly mediate the mother’s behavior toward her child (Cabrera et al., 2018). When mothers perceive their co-parenting relationship positively, infants’ negative affect and stress reactivity decrease (Belsky et al., 1991) and soothability increases (Feinberg and Kan, 2008). Increased father involvement has been associated with decreased maternal intrusiveness during play and increased exploratory play by the child (Feldman et al., 1997). Yet, there is still much to learn about exactly how fathers impact maternal care characteristics and vice versa. Father care may be impacted in similar ways to mother care and is potentially mediated by similar neural mechanisms. Both mothers and fathers suffering from depression show reduced positive parenting behaviors (Wilson and Durbin, 2010). Fathers also show changes in oxytocin hormone following the birth of a child, just as mothers do, and the magnitude of these changes does not differ based on parental sex (Gordon et al., 2010; Grumi et al., 2021). A few studies have also investigated the impact of each individual’s parenting behavior on the other parent’s parenting behavior. In families where mothers suffer from postpartum depression, fathers may become more involved in caregiving (Edhborg et al., 2003; de Mendonça et al., 2012). Parents also show a negative association in engagement, such that when one parent is more engaged with the child, the other parent’s level of engagement is reduced (Li et al., 2022). These behavioral patterns have been termed “compensatory.” However, other work has shown that maternal depression may worsen the father-child relationship (Goodman, 2008), or that more sensitive fathers help mediate the impact of maternal depression on the relationship to the child (Muzard et al., 2021). These disparate results may reflect the complexity of variables impacting the parent-child triad, but together suggest that the parenting behavior of each partner is susceptible to that of the other. The extent to which parenting by one impacts parenting by the other remains unclear.

While animal models of parenting present their own challenges to interpretation, such methods provide a complementary perspective to human parenting research by addressing some of the research barriers that are difficult to overcome in human-based projects (Bales, 2017). Animal models are particularly well-suited to isolating specific behavioral changes, by removing many of the other environmental variables that might impact the results. There is a well-established body of research exploring maternal behavior in common laboratory species, such as mice and rats. For example, variation in the amount of maternal care a rat provides can have lasting impacts on her offspring’s social and anxiety behaviors (Caldji et al., 1998; Champagne et al., 2003; Parent et al., 2013). However, biparental species are less common targets for parenting research and father-care likely impacts overall parenting in highly relevant ways. The prairie vole (*Microtus ochrograster*) is a biparental and socially monogamous rodent, one of the small percentage of all mammalian species where fathers provide parental care (Getz et al., 1981). Maternal, paternal and combined dyadic care exhibit a naturally occurring range of behaviors (Perkeybile et al., 2013). Several of these naturally varying species typical behaviors are also sensitive to early life manipulations. Vole offspring raised by a single mother will exhibit delays in species typical partner preference formation and decreases in spontaneous alloparental behavior (Ahern and Young, 2009; Ahern et al., 2011). Female offspring raised by high-care pairs have increased juvenile affiliation and will spend more time with their opposite-sex partner (Perkeybile et al., 2013; Arias del Razo and Bales, 2016). These experiments illustrate that the quality of parental care is critical to the social development of offspring in biparental species, but the origins of natural parenting variation, particularly the impact of father-care on the parenting dyad remains unclear. Given the high utility of the prairie vole as a complementary model species in understanding early life development, it is critical to establish how prairie voles learn to parent as part of this parenting dyad.

In this study, we investigate how naive prairie voles learn to be parents. Learning, as a concept, has been defined in multiple ways over time and across sub-fields. This has sometimes introduced interpretive problems when competing or unclear definitions are offered (Barron et al., 2015). Here, we operationalize the term “learning” to mean changes in behavior associated with life experience, and we make no assumptions about the specific cognitive mechanisms by which this behavioral change occurs. The effect of a specific life experience on parental behavior was assessed: the experience of parenting as part of a dyad. To do this, we paired unrelated male and female voles and observed care levels provided to their own offspring in their first two litters. This allowed for the comparison of behavior between the naive (first litter) and experienced (second litter) parenting contexts in the same sets of parents. By observing two subsequent litters, we are better able to understand how parental behavior develops in a pair. We focused our care observations on the first 3 days post birth as prior work has shown this to be a critical period in which differences in parental care are most associated with later life offspring social differences (Bales et al., 2011; Perkeybile et al., 2013).

If parental care in the prairie vole is highly stereotyped, we would expect to see minimal variation between individuals and within sets of parents. However, prior work indicates that there is substantial variability in the care provided by different parenting dyads (Perkeybile et al., 2013; Finton and Ophir, 2020). Given this variability, we might also expect little change across litters if such behaviors are highly genetically determined or determined only by early life experiences. Whether such changes occur over time has not been definitively established in the prairie vole, as there is minimal and conflicting literature with some evidence stating vole parental care remains stable between litters (Ahern et al., 2011; Perkeybile et al., 2013), and some evidence stating it may vary between litters (Rogers et al., 2018). However, if it is the case that parenting behavior adjusts over the course of time, this indicates that parenting behavior is dynamic and may be influenced by aspects of the current environment. We question whether care behavior by one parent will be influenced by that of the other parent. There is little extant literature examining this dynamic as much of the work claiming stability between litters was done by examining changes in established pairs and focusing on the combined care of the dyad. As such, we propose two alternative potential outcomes based on the human parenting findings we have previously outlined, namely that vole parents may show a “compensatory” pattern of behavior, in which lower care from one parent is associated with higher care from the other parent, or that parental behavior may show an “enhancement” pattern of positively correlated behavior, such that lower care from one parent is associated with lower care from the other parent. Importantly, this study seeks to provide some clarity to these conflicting predictions.

## 2 Materials and methods

### 2.1 Animal model

Laboratory-bred prairie voles (*Microtus ochrogaster*) were maintained on a 14:10 light-dark cycle with lights on at 0700 during daylight savings and 0600 the rest of the year. Animals were given food (high-fiber Purina rabbit chow) and water ad libitum with cotton nestlets for nesting material. After weaning on postnatal day 20, same sex sibling pairs were housed in small polycarbonate cages (27 cm × 16 cm × 16 cm). Breeding pairs were housed in larger polycarbonate cages (44 cm × 22 cm × 16 cm). All procedures were reviewed and approved by the University of Virginia’s Institutional Animal Care and Use Committee.

### 2.2 Creation of experimental pairs

Over the course of 2 years, 137 sexually and experimentally naive breeder pairs aged post-natal day (PND) 61 to PND132 were created for use in multiple experiments in our lab examining the impacts of parenting behavior. Typical vole gestation is 21.5 days, so 20 days after mating, pairs were moved into a clean cage, and a small line of fur was trimmed across the males’ hips as an identifier for behavioral scoring. Cages were checked daily to monitor for pups, with the date of birth being PND0. Litter sizes were not standardized, as there is a body of literature highlighting that early life handling leads to an increase in parenting behavior (Perkeybile et al., 2019). It has been previously shown that there is no significant relationship between amount of total parental care and litter size (Danoff et al., 2023). Using Pearson’s R, we replicated this with our own sets of parents, finding no significant relationship between the total (*R* = 0.040, *p* = 0.56), male (*R* = 0.01, *p* = 0.88) or female (*R* = 0.039, *p* = 0.57) care provided and the number of offspring in the litter. Of the original 137 breeding pairs, 22 pairs were eliminated as they never produced a litter. Five additional pairs were eventually dropped, as they did not produce a second litter, or one of the parents died for unknown reasons. The final number of adult animals used in these analyses is 220 animals in 110 parental dyads.

### 2.3 Measurement of prairie vole parenting

**FIGURE 1:**
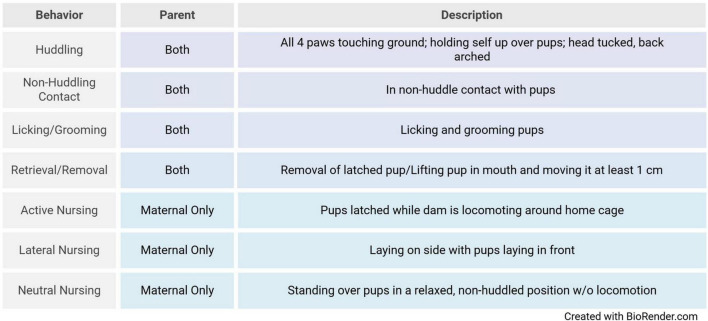
Behavioral ethogram of parental behaviors. Descriptions of parental, and maternal nursing behaviors observed during parental care quantification and observations [adapted from Perkeybile et al. (2013)].

Parenting behavior assessment in the prairie vole was performed using previously established methods [see (Perkeybile et al., 2013)]. Briefly, parenting behavior was live scored in the home cage by a trained observer using behavioral software^1^. Observers were blind to the history of animals they were scoring. Animals were not disturbed during the observation period. The behaviors of both the mother and father were simultaneously scored. The scored behaviors are listed in Figure 1. Observations occurred a total of four times per litter for 20 min each. All observations were made between PND1 and PND3, with two observations occurring in the morning and two observations occurring in the afternoon. Observations were completed for litters 1 (naive) and 2 (experienced). Mean duration of pup directed behaviors were summed for each litter to produce (1) a maternal care score, (2) a paternal care score, and (3) a total care score for each litter, see Figure 1.

### 2.4 Statistical analysis and graphs

Analyses of parenting behavior were performed in RStudio (Posit team, 2025) version 4.3.3 (R Core Team, 2024), and final figures were made using BioRender (Hinton, 2025a,b) and the matplotlib package in Python (Hunter, 2007). Parenting behavior was assessed using mixed effects linear models in the “lme4” package (Bates et al., 2015), with *p*-values for factors derived from two-tailed Type-II Wald ^2 tests via the “Anova” function in the “car” package (Fox and Weisberg, 2019). *Post-hoc p*-values and contrasts were extracted using the “emmeans” package by first calculating the estimated marginal means, then preforming pairwise comparisons for tukey adjusted *p*-values (Lenth, 2024). Effect sizes and explained variances were calculated using the “rstatix” (Kassambara, 2023) and “MuMIn” (Bartoń, 2024) packages, respectively.

## 3 Results

### 3.1 Group-wise analyses

#### 3.1.1 With experience, parents provide less care

**FIGURE 2:**
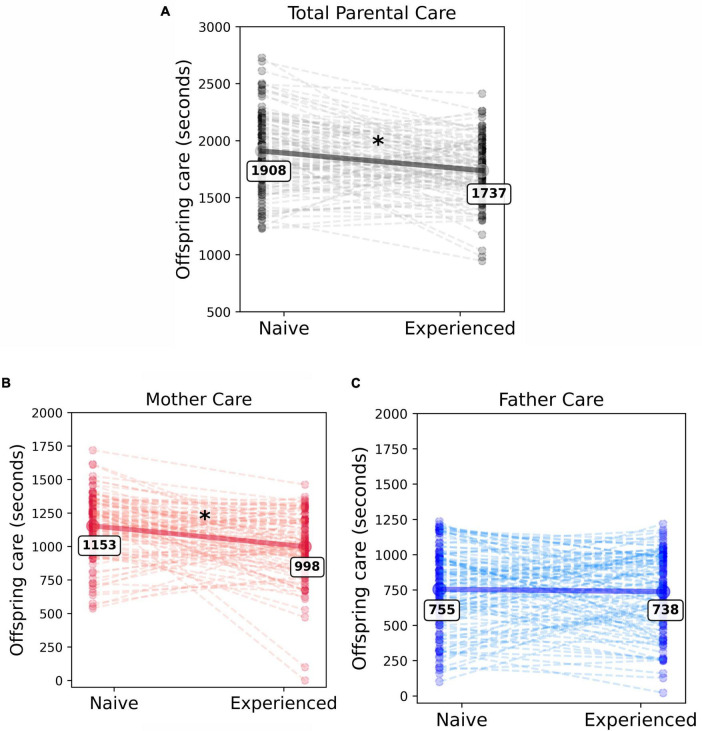
Experience-dependent reduction in total parental care is driven by reductions in mothers care. Individual dots represent each unique parent dyad **(A)**, individual mother **(B)** or individual father **(C)**. The dashed lines connect the same dyad or individual’s behavior between the naive and experienced contexts. **(A)** The decline in total care (sum of maternal and paternal care) provided to offspring with experience was significant (χ^2^_(1)_ = 27.85, *p* < 0.001). **(B)** Mothers provided more care than fathers in both the naive and experienced contexts, however, they significantly reduced their care investment when experienced. **(C)** As a group, fathers did not significantly alter the amount of care they provided with experience. * Denotes significance.

To determine whether the experience of parenting changes total parenting behavior, a linear mixed-effects model was used. Total parental care, defined as the sum of both maternal and paternal care provided to pups, was the model’s dependent variable. Context (naive versus experienced parenting) was the sole fixed effect. Parent dyad was defined as a random effect to account for the potential that each dyad has some unique amount of parental care consistent across multiple litters of pups (i.e., a tendency to provide a similar amount of care regardless of context). This seemed likely given prior reports of consistently high or low care parental dyads across multiple litters. We found that the total care behavior was significantly different between contexts [β = −171.14, SE = 32.37, *t*_(109)_ = −5.29, *p* < 0.0001]. Care was significantly reduced from the naive context (mean = 1908.75, SD = 336.42) to the experienced context (mean = 1737.61, SD = 273.03). Figure 2A demonstrates this decrease in total care with experience. On average, parents reduced their care effort by 171 seconds, or about 9%, when experienced. When comparing the relative impact of context and individual parental dyad, we found that 7.26% (*R^2^m* = 0.0726) of the variation in total parental care was explained by the effect of context alone, while the inclusion of parent dyad as a random effect increases the model’s explained variance to 43.06% (*R^2^c* = 0.4306). While the context in which the parental care was provided accounts for a significant amount of the variance in total parental care, the tendency for each parent dyad to provide a stable amount of care across litters substantially increases the model’s explanatory power. Thus, the identity of the dyad is relevant and should be considered when assessing potential contributions to parental care behavior.

#### 3.1.2 Maternal behavior is the driver of care reduction

While the previous model indicated that parent dyads decreased total care overall with experience, it did not differentiate between the care provided by the mother and the father. To assess whether both mothers and fathers contributed to the observed decrease in total care, we ran a second linear mixed-effects model to understand how maternal care and paternal care changed independently from the naive to experienced contexts. The individual care contribution (not the total contribution) was the dependent variable. As in the total care model, context (naive versus experienced) was a fixed effect. Sex was added as an additional fixed effect. A random effect was included to account for any potential consistency of each individual parent included in the model across the multiple observations of parenting. We did not include parent dyad as an additional random effect because we wanted to demonstrate the impact of modeling each individual alone without reference to the parent dyad.

The model reported that the main effect of sex was significant (β = −398.56, SE = 35.32, *t*_(398.14)_ = −11.28, *p* < 0.0001). On average, mothers provided significantly more care than fathers in both naive and experienced contexts. Mothers provided a mean of 1,076 s of care, while fathers provided 747 s of care, an approximately 59:41 proportion of care effort from mothers and fathers, respectively. There was substantial variability in care provided by mothers (range = 1.0–1719.0, SD = 250.17) and fathers (range = 22.0–1235.25, SD = 281.94), indicating that care behaviors are not highly stereotyped across all individuals. The model also reported that the fixed effect of context was significant [β = −154.87, SE = 29.38, *t*_(218)_ = −5.27, *p* < 0.0001]. On average, an individual parent reduced the care provided to his or her offspring by 85.57 s from the naive to experienced contexts. The average naive parent provided 954.38 s of care (SD = 329.55), while the average experienced parent provided 868.80 s of care (SD = 289.85).

We found a significant interaction between context and the sex of the individual parent [β = 138.59, SE = 41.55, *t*_(218)_ = 3.34, *p* = 0.001]. Mothers provided significantly more care as naive parents when compared to their experienced context [*t*_(218)_ = 5.27, *p* < 0.001; naive: mean = 1153.65, SD = 233.46; experienced: mean = 998.79, SD = 244.37; Figure 2B]. Fathers’ care did not significantly change across contexts [*t*_(218)_ = 0.55, *p* = 0.58; naive: mean = 755.097, SD = 290.771; experienced: mean = 738.820, SD = 275.234; Figure 2C]. Additional post-hoc testing indicated that while naive mothers provided an average of 399 s more care than naive fathers [estimated marginal means (emm) = 399, SE = 35.3, *t*_(398)_ = 11.28, *p* < 0.0001], this difference is reduced in the experienced context. While experienced mothers still provide significantly more care than experienced fathers, mothers provided only 260 more seconds of care than fathers, a reduction of 139 s from the naive context [emm = 260, SE = 35.3, *t*_(398)_ = 7.36, *p* < 0.0001]. The typical proportion of total care provided by mothers decreases from nearly 61% in the naive context to approximately 57.5% in the experienced context, indicating that a pattern of more equal distribution of care develops between the two sexes with experience. Context, sex, and their interaction explained 30.55% (*R^2^m* = 0.3055) of the variation in individual care contribution. The inclusion of the random effect accounting for individual parent consistency increased the explained variance of the model to 51.97% (*R^2^c* = 0.5197). This indicates that while individual parent identity is a relevant predictor in the model, solely knowing the context and sex of the parent provides a large proportion of the model’s predictive power.

We next sought to understand if the reduction in mothers parental care was driven by one or more of the specific behaviors we measured (see Figure 1 for the full list). For both mothers and fathers we ran separate linear mixed effects models for each of our parental behaviors, with context as a fixed effect and a random effect of ID to account for multiple observations of parenting. Experienced fathers spent significantly more time huddling, and significantly less time in non-huddle contact with their offspring, all other behaviors were not significantly different between contexts (see Supplementary Figure 1 for means, standard deviations, and statistical reporting on all behaviors). The experience-based reduction in total maternal care was not driven by any one behavior, but by a general reduction in many of the observed behaviors. Group wise, experienced mothers showed a statistically significant reduction in time spent huddling, licking and grooming, and neutral nursing their second litter of pups, and an increase in time spent lateral nursing (Supplementary Figure 1).

### 3.2 Dyadic analyses

#### 3.2.1 Maternal behavior becomes more negatively correlated with her male partner with experience

**FIGURE 3:**
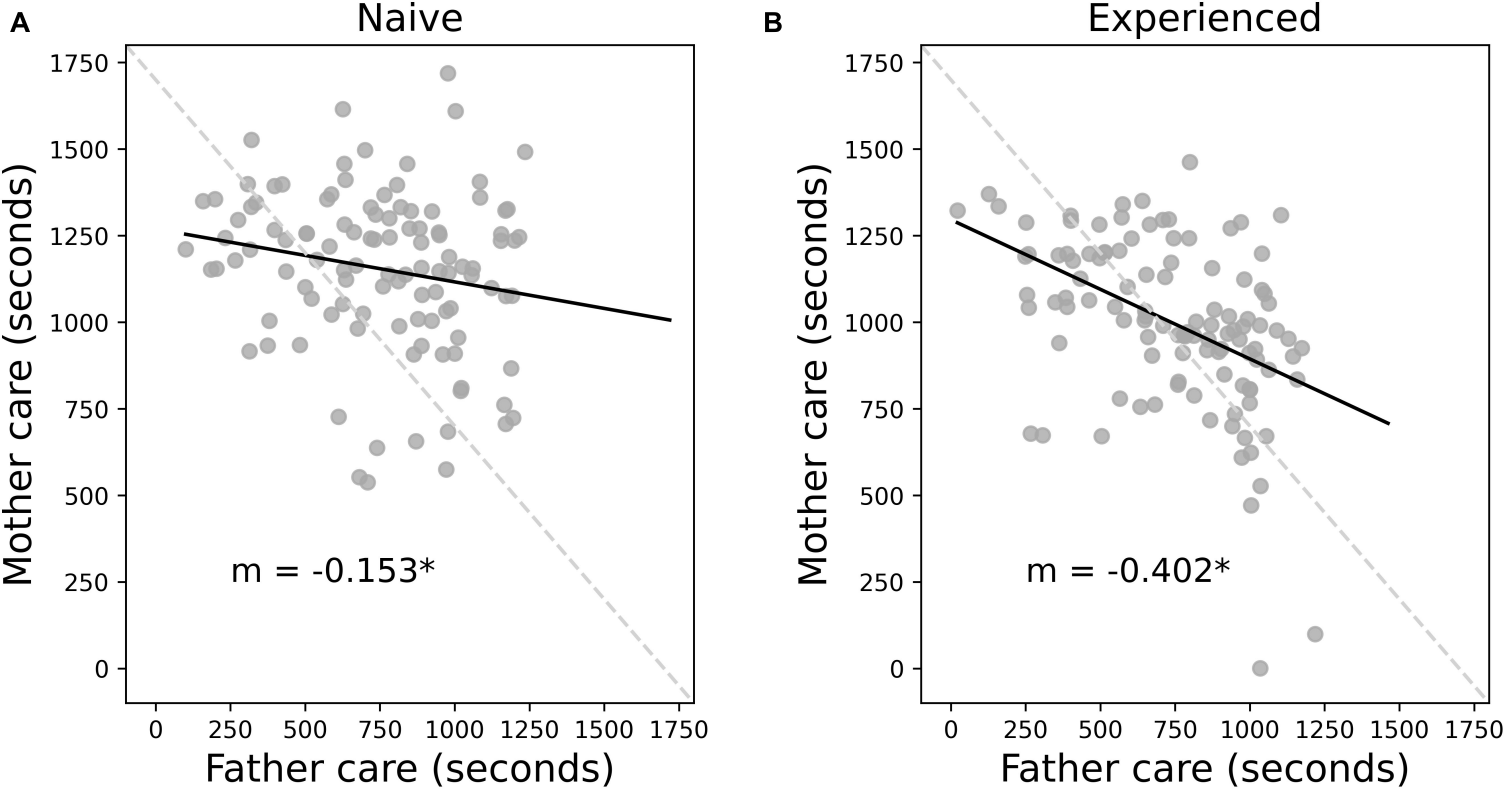
Negatively correlated care behavior between mothers and fathers becomes more pronounced with experience. Regardless of context, fathers’ care level significantly negatively predicts mothers’ care level. This is true in both the naive **(A)** and experienced **(B)** contexts. A typical higher care mother is more likely to be associated with a lower care father and vice versa. Individual parent dyads are plotted in gray, and the trend in the relationship between mother and father care is shown with solid black lines. In both contexts, the slope of the trend line (*m*) is negative (*m* = –0.152 and *m* = –0.402 for naive and experienced contexts, respectively), indicating a negative correlation in maternal and paternal care. The dark gray dashed line shows a perfect negative correlation. The shallower actual trend line indicates an asymmetry in the male-female care association, such that mothers tend to provide a higher level of care overall even when paired with higher care fathers, though this asymmetry is reduced with experience. There was a significant interaction between context and the effect of paternal care on maternal care. The slope of the experienced maternal-paternal care relationship is significantly more negative than the naive relationship, showing that higher care in mothers is more associated with lower care in fathers in the experienced context. * Indicates statistical significance *p* < 0.05.

The prior analyses indicated that total care was reduced overall with experience and appeared to suggest that this reduction in care was driven by reduced female care alone. However, these group-wise analyses did not account for the changes in care within specific parent dyads. By treating each parent as a separate individual, it remains unclear whether mothers are adjusting their behavior to that of their specific male partner, or whether changes in care are independent of their male partner’s behavior. In the case of independent change, we would expect to see that a mother decreases her care investment with experience regardless of her male partner’s care behavior. To better understand this dynamic, an additional linear mixed-effects model was implemented with mothers’ individual care as the dependent variable. As before we used context (naive versus experienced) as our first fixed effect, with fathers’ individual care as a second fixed effect. A random effect of parent dyad was included to account for potential care consistency within each set of parents. Context alone was not significant [β = 23.54, SE = 79.36, *t*_(154.62)_ = 0.29, *p* = 0.77], but fathers’ individual care [β = −0.17, SE = 0.07, *t*_(210.27)_ = −2.40, *p* = 0.017] and the interaction between fathers’ care and context [β = −0.25, SE = 0.10, *t*_(161.36)_ = −2.45, *p* = 0.015] were both significant. A *post hoc* analysis showed that a father’s behavior negatively predicts the mother’s behavior in both the naive [β = −0.17, SE = 0.07, *t*_(210)_ = −2.38, *p* = 0.018; Figure 3A], and experienced [β = −0.42, SE = 0.08, *t*_(210)_ = −5.44, *p* < 0.0001; Figure 3B] contexts. However, the slope of the negative correlation became significantly steeper in the experienced context [SE = 0.081, *t*_(320)_ = 2.91, *p* = 0.0038], indicating that a mother’s care investment becomes more negatively correlated with her male partner’s care investment when the parent dyad is experienced. Thus, the father’s care level becomes more predictive of his female partner’s behavior with experience, such that a low care investment father is more likely to be paired with a high care investment mother, compared to the naive context. Context, fathers’ care, and their interaction explained 21.59% (*R^2^m* = 0.2159) of the variation in maternal care contribution. The inclusion of the random effect of parent dyad increased the explained variance to 39.79% (*R^2^c* = 0.3979).

#### 3.2.2 Dynamic duos: parents adjust their care together

**FIGURE 4:**
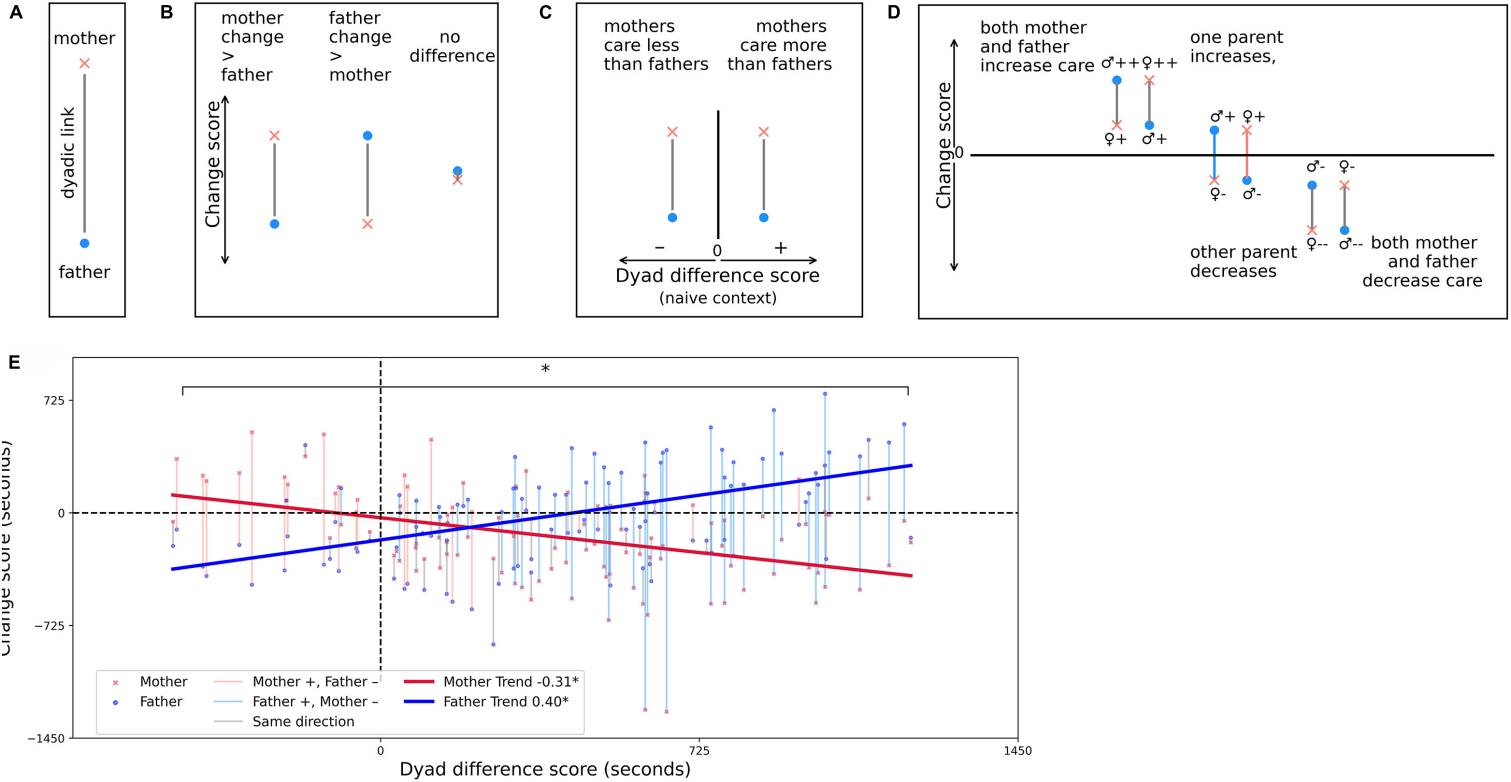
Larger care discrepancies between mothers and fathers in the naive context result in larger shifts in care with experience. Regardless of sex, a relatively lower care naive partner is more likely to increase his or her care, while a higher care partner is more likely to decrease care with experience. **(A–D)** Subplots are schematic renderings to aid in interpreting **(E)**. **(A)** Mothers and fathers are represented by red x’s and blue circles, respectively. Parent dyads are always represented as a dyad with their dyadic link shown as a connecting line between the two symbols. **(B)** The change score (CS_i_), a measure of the change between the naive and experienced contexts, is represented on the y-axis. A higher red x symbol on the y-axis indicates that mothers increased their care more than fathers, while a higher blue dot symbol indicates that fathers increased their care more than mothers. Overlapping symbols indicate that mothers and fathers changed the same amount. **(C)** The dyad difference score (DS_p_naive_), a measure of the magnitude of difference between mothers and fathers in the naive context is displayed on the x-axis. If the dyad is plotted to the left of X = 0, the mother provided less care than her male partner (*N* = 18 instances). If the dyad is plotted to the right of X = 0, the mother provided more care than her male partner (*N* = 92 instances). **(D)** Three possible CS_i_ scenarios are possible. At left, Y < 0, both the mother and father in the dyad increased their care in the experienced context. Depending on which symbol is higher, either mother (red x) or father (blue dot) increased more than their opposite sex partner. At right, Y > 0, the opposite scenario is displayed, in which both mother and father decreased their care. At the center, where the line crosses 0, one parent increased his or her care, while their opposite sex partner decreased their care. This was by far the most common scenario observed (*N* = 70). Dyadic links are color coded according to the pattern of observed change in both parents: gray line = both parents increase or decrease, blue line = male increase, female decrease, red line = female increase, male decrease. **(E)** 110 parent dyads are plotted. The solid red line shows that the female CS_i_ was significantly negatively associated with the DS_p_naive_. The solid blue line shows that the male CS_i_ was significantly positively associated with the DS_p_naive_. The opposite pattern of effects is reflected in the significant interaction between sex and DS_p_naive_ reported by the model. Given that DS_p_naive_ imposes a more negative score on higher care fathers and a more positive score on higher care mothers, these opposite going trend lines actually reflect the same pattern of behavior across both sexes. Namely, providing relatively higher care than one’s partner in the naive context makes it likely that an individual will reduce his or her care when experienced, while their relatively lower care partner is likely to increase his or her care. * Denotes significance.

The prior model helps demonstrate that adjustments in maternal behavior with experience are influenced by her male partner’s behavior. However, this approach presupposes that only mothers adjust to their partner’s behavior. While fathers do not, on average, appear to change care behavior between contexts, we observed that a subset of fathers appeared to increase or decrease their care (evident in Figure 2C). It seemed possible that, while there were no group-wide changes in paternal care, there might be some factor that accounted for an individual father increasing or decreasing his care with experience. To mitigate this gap in understanding, we performed a final analysis. We tested whether the adjustment in care each individual parent provided between the naive and experienced contexts, termed the change score (CS_*i*_) could be predicted based on the discrepancy in care provided by each parent within the dyad in the naive context, termed the dyad difference score (DS_p_naive_). Equations 1.1, 1.2 demonstrate these calculations. A positive DS_p_naive_ indicates that mothers provided relatively more care than fathers in the naive context, while a negative DS_p_naive_ indicates that naive fathers provided relatively more care (for example, see Figures 4A–D). We theorized that parents who exhibited relatively larger discrepancies in care efforts in the naive context might adjust their care more with experience than those whose partnership already displayed more equal amounts of effort.

***Equation 1. Measures of parental care adjustment and difference in naive parental care.***


Definitions⁢of⁢terms:

Scores:Change⁢score⁢(CS),Dyad⁢difference⁢score⁢(DS)

Sex:male⁢(m),female⁢(f)

Unit⁢of⁢measurement:individual⁢(i),parent⁢dyad⁢(p)

$$\mathrm{Equation}\,1.1\;C\,S_{i}=c\,a\,r\,e_{e\,x\,p\,e\,r\,i\,e\,n\,c\,e\,d}-c\,a\,r\,e_{n\,a\,i\,v\,e}$$

$$\mathrm{Equation}\,1.2\;D\,S_{p\,\_\,n\,a\,i\,v\,e}=c\,a\,r\,e_{f\,\_\,n\,a\,i\,v\,e}-c\,a\,r\,e_{m\,\_\,n\,a\,i\,v\,e}$$

To understand the relationship between DS_p_naive_ and CS_i_, a two-way analysis of variance (ANOVA) model was conducted. CS_i_ was the dependent variable. The DS_p_naive_ and sex were the independent variables. The interaction of sex and DS_p_naive_ was also included in the model. There was no significant main effect of DS_p_naive_ [*F*_(1, 216)_ = 0.93, *p* = 0.34], however there was a significant main effect of sex [*F*_(1, 216)_ = 14.14, *p* < 0.001]. This effect is analogous to the previous finding that females change more than males from naive to experienced contexts. More notably, there was also a significant interaction between sex and DS_p_naive_ on CS_i_ [*F*_(1, 216)_ = 60.03, *p* < 0.001, ηp^2^ = 0.22], indicating a large effect. Post-hoc testing indicated a significant difference in the slopes of the relationship between CS_i_ and DS_p_naive_ between mothers and fathers [β = −139, SE = 36.9, *t*_(216)_ = −3.76, *p* = 0.0002], such that as DS_p_naive_ scores increase, female CS_i_ significantly decreases [β = −0.309, SE = 0.065, *t*_(216)_ = −4.80, *p* < 0.0001], and male CS_i_ significantly increases [β = 0.397, SE = 0.065, *t*_(216)_ = 6.16, *p* < 0.0001, Figure 4E]. This indicates that experienced mothers and fathers adjust their care based on their past experience with their opposite-sex partner. Given that the DS_p_naive_ is sex-directional, the opposite-direction trend lines for male and female parents indicate that differences in the naive context result in the same directional impact for both males and females– that is, a larger discrepancy in care between partners in the naive context, the relatively lower care partner will *increase* their care efforts in the experienced context, while the relatively higher care partner will *decrease* their contribution, regardless of sex. While fathers may not show a significant change in care investment overall, those whose female partner provided relatively less care than he did in the naive context were the most likely to decrease their care investment with experience, while their female partner was more likely than other mothers to increase her investment. Thus, the significant interaction between sex and prior experience is driven by adjustments in care made by both parents.

## 4 Discussion

Deploying novel dyadic analyses reveals a previously hidden dynamic between male and female prairie vole parents. By comparing the results of traditional group-wise analyses with a dyadic analysis, we show these subtle dynamic behavioral changes; not only are mothers responsive to the specific behavior of their male partner, but this responsiveness is mutual. Fathers are not passive bystanders and can adjust their behavior based on their experiences with their female partner. As reported in prior prairie vole literature (Rogers et al., 2018), we found that dyads significantly reduced the total care they provided to offspring as they became experienced parents. Sets of parents provided highly variable amounts of care to their offspring, indicating that care amounts are not tightly stereotyped. The high amount of variability in the model explained by the parent dyad as a unique pair regardless of context suggested that the unique characteristics of each dyad’s parental care investment remains somewhat stable over time. For example, while our results show that, overall, parents tend to reduce care with experience, a higher care dyad in the naive context is likely to remain a relatively high care dyad in the experienced context.

When investigating differences between mothers and fathers in level of care investment, we found that the typical mother provides more care than the typical father in both the naive and experienced context. This difference in care is reduced with experience, with mothers, on average, decreasing their individual care and looking more father-like. Thus, the overall decrease in total care with experience that we observed appeared to be driven largely by reductions in group-wide maternal care. Rogers et al. (2018) previously found a similar pattern of behavior and proposed that this decline may be due to mothers becoming more efficient with their care or less sensitive to cues from pups (Rogers et al., 2018). Our findings that mothers reduce many of their parental care behaviors from naïve to experienced provide additional evidence for this argument. In human literature, parents also appear to invest more resources, such as quality time (Keller and Zach, 2002; Price, 2008), and prenatal and preventative health care (Buckles and Kolka, 2014; Pruckner et al., 2021), in their firstborn children.

Without further investigation, it is impossible to be sure whether decreases in care are equally typical of all mothers, or whether mothers might adjust their care based on their unique experience with their male partner. These group-wide findings might lead one to conclude that father care is relatively fixed and unchanging based on learned parenting experience. However, as we inspected our data, we noticed that some fathers did appear to be increasing or decreasing their care investment with experience. We wondered whether there might be some explanation for this variability in fathers’ changing care patterns that was masked by the more dramatic and clear-cut maternal downshift in care effort. To address these questions, we embarked on a series of two analyses which attempted to more specifically take into account the interplay between the two parents, rather than treating their individual behavior as completely independent from their partner’s behavior.

With this approach, we found that maternal care behavior becomes more negatively correlated with her male partner’s behavior with experience. This may be, in part, because most mothers (84% of all mothers) provided more care than their male partner in the naive context. As mothers are required for the early nutrition and survival of the offspring, greater maternal care is expected overall. However, the level of care provided by their male partner helps to explain the magnitude of the reduction in care a mother was able to make. For example, a very high care mother is likely to reduce the care she provides with experience regardless of her male partner’s behavior, but will reduce her care less if the male partner provides relatively little care effort. The initial burst of mothering effort in the naive context slightly obscures a more enduring pattern of negatively correlated care behavior in experienced parent dyads, such that higher care mothers are more likely to be associated with lower care fathers, and vice versa.

While we did not make any assumptions about the cognitive processes underlying changes in care behavior between the naive and experienced contexts, we considered that something about the quality of the experiences gained by the vole parents could explain how they changed their care investments. By employing the difference in care provided by male and female partners in the naive context as a predictor of change in care, we showed that naive parent dyads with larger inter-parent discrepancies in care behavior changed their care investments more in the experienced context. Importantly, this finding applied to both the mother and the father within the dyad. While less typical, 18 of the 110 mothers provided less care than their male partner in the naive litter. Within this subset, mothers tended to increase their care with experience, while their higher care male partner tended to decrease his care. These lower care mothers show that the same dynamic that applies to mothers in the more common scenario in which she is the higher care partner also applies in the less common scenario in which the father is the higher care partner. Namely, whichever parent provided less care in the naive context is likely to increase their investment in the experienced context, while their partner will decrease his or her investment. Ultimately, this dynamic shifting between the parents explains the reduction in care differences we initially observed, in which the proportion of care provided by each parent becomes more equal with experience (though still skewed towards higher maternal care). This suggests that sensitivity to offspring’s needs and efficient parenting investment is less sex-specific than suggested by prior work. It is possible that these findings represent an ongoing dynamic process that may not be tied solely to the process of gaining parental experience. Further work might investigate this by tracking pairs for longer to observe whether the dynamic we identified remains stable or continues to shift in subsequent litters.

We speculate that vole fathers play a special role in shaping the parenting behavior of their partners future parenting. What are fathers for and why do prairie voles, unlike most other mammal species, generally utilize father care to raise young? Previous work has indicated that when the father is removed, vole mothers do not adjust the amount of care they provide to their young to compensate for the missing father (Ahern and Young, 2009; Ahern et al., 2011; Hiura et al., 2023). While offspring of these mothers do not generally die, and are capable of forming social partner bonds later in life, it does take them longer to do so than those raised by both parents (Ahern and Young, 2009). This suggests that the contributions of the father are beneficial in increasing offspring fertility, which fits with the evolutionary theory that paternal care behavior becomes adaptive when the amount of care provided by mothers alone is not sufficient to adequately provision existing offspring or maintain a certain level of fertility (Hrdy, 2009). Additionally, prior work from our lab shows that much of the variability in parental care provided by the breeder pair overall is determined by the amount of care the father invests (Danoff et al., 2023). Taken together, these findings suggest that maternal care behavior is likely already at or close to a ceiling. In this work, we only saw mothers adjusting their care investment upwards when they were providing less care than average. Since fathers seem to be a greater source of variability in care received by offspring than mothers, father care may appear to have an outsized effect on offspring’s future behavior. Given an absent mother, pups are unlikely to survive. If the mother is present, she tends to provide a fairly consistent and potentially at- or close-to-ceiling amount of care, such that her offspring are not heavily impacted by any small deviations in care. However, given an absent father, pups may show social delays and differences in adult behaviors.

We operationalized the term “learning” to indicate any observed changes in parenting investment from the naive to experienced context. However, one could argue that the mechanisms of this change are very simple. For example, dyads may show this “compensatory” pattern is simply due to shifts in maternal care alone, with father care merely reactionary, as a father cannot provide high levels of care if his partner is huddled over pups and preventing access. Based on our anecdotal observations, this does not seem to be the case, as fathers will readily huddle next to the mother and pups in the nest, maintaining some contact with the pups despite the maternal huddling behavior. Additionally, we have observed some cases in which either a father or mother will “retrieve” their partner when they are ready to switch places in the nest by seeking out the partner and either touching noses with them or, more forcefully, attempting to drag the partner into the nest [a behavior that has previously been reported by Ahern and Young (2009)]. These behaviors, while anecdotal, are suggestive that even the relatively simple prairie vole is able to deploy some sophisticated behaviors to balance parenting duties across partners.

Future work could further investigate how parents adapt their parenting style to each other by re-pairing each parent and seeing if their behavior adjusts to a new partner or if they exhibit behaviors closer to how they parented before. Our models only accounted for around 50% of the variance seen in parental behavior. As seen in other rodent models of parental care, we suspect that additional variance may be due intergenerational behavioral inheritance. Cross fostering work in rats has indicated that maternal behavior is passed from mother to daughter via non-genomic means (Weaver et al., 2004). Future projects could utilize a similar cross-fostering paradigm in the prairie vole to examine the additional impacts early life parental care received may have on the development of an individual’s own parenting. Our results here suggest that vole parenting behaviors may be plastic and very susceptible to environmental factors. We also showed that parenting in a dyad and having a father introduces a large amount of variance into the amount of care offspring receive and it should be taken into consideration when looking at how the early life environment may impact outcomes in offspring.

## Data Availability

The raw data supporting the conclusions of this article will be made available by the authors, without undue reservation.

## Footnotes

1
www.behaviortracker.com
