# Supplementary figures and images for: Dynamic duos: learning to care as a pair in the biparental prairie vole (Microtus ochrogaster)
Source: Front Behav Neurosci. 2025 Nov 26;19:1698616. doi: 10.3389/fnbeh.2025.1698616 (PMC12689902; doi:10.3389/fnbeh.2025.1698616)

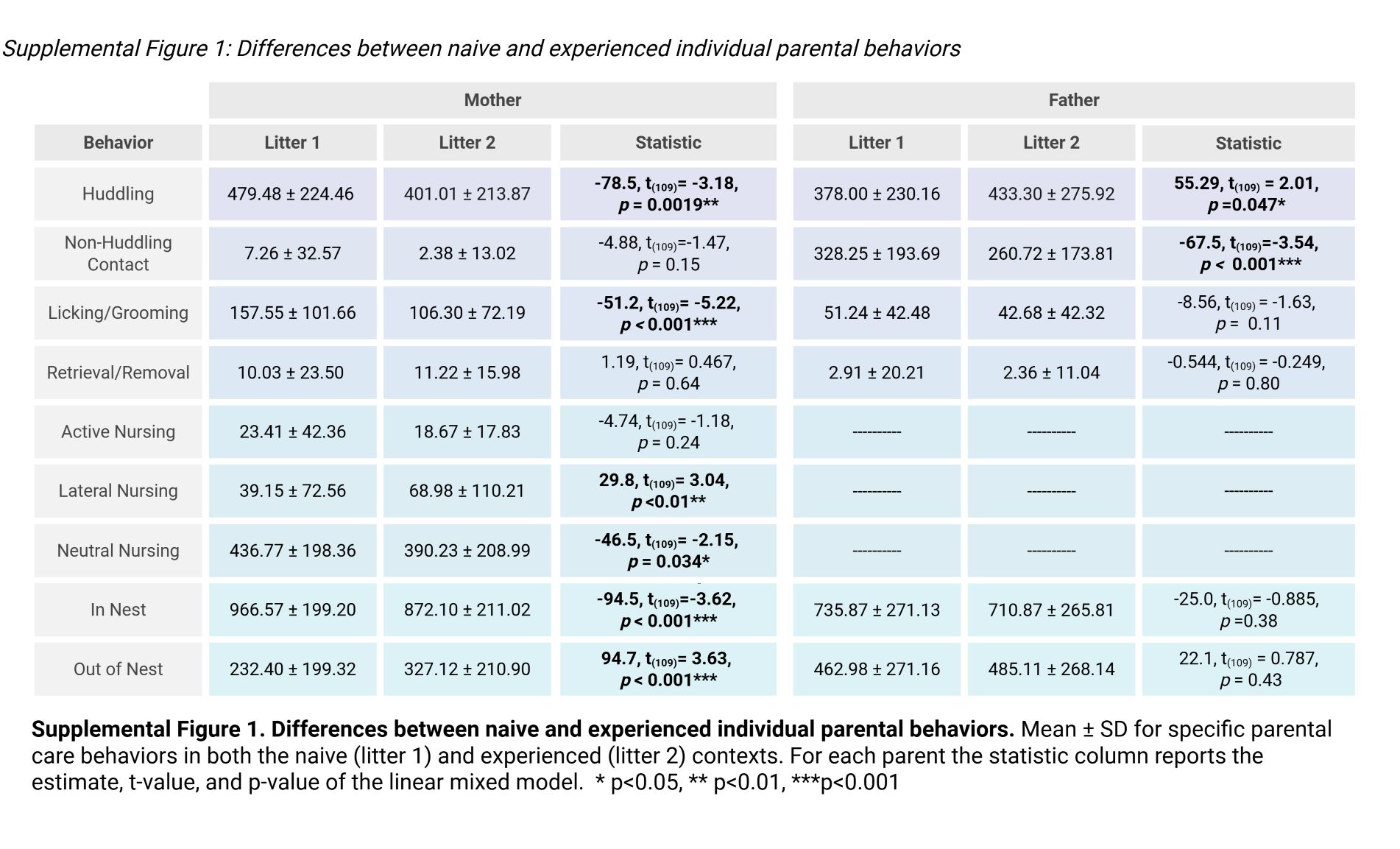

Supplement: Supplementary file 1 [file Image_1.jpeg]
